# Repetition Is the Feature Behind the Attentional Bias for Recognizing Threatening Patterns

**DOI:** 10.1177/1474704918754782

**Published:** 2018-02-18

**Authors:** Maryam Shabbir, Adelynn M. Y. Zon, Vivek Thuppil

**Affiliations:** 1 School of Psychology, University of Nottingham Malaysia Campus, Semenyih, Selangor, Malaysia

**Keywords:** attentional bias, visual attention, feature-based attention, provocative patterns, repetitive patterns

## Abstract

Animals attend to what is relevant in order to behave in an effective manner and succeed in their environments. In several nonhuman species, there is an evolved bias for attending to patterns indicative of threats in the natural environment such as dangerous animals. Because skins of many dangerous animals are typically repetitive, we propose that repetition is the key feature enabling recognition of evolutionarily important threats. The current study consists of two experiments where we measured participants’ reactions to pictures of male and female models wearing clothing of various repeating (leopard skin, snakeskin, and floral print) and nonrepeating (camouflage, shiny, and plain) patterns. In Experiment 1, when models wearing patterns were presented side by side with total fixation duration as the measure, the repeating floral pattern was the most provocative, with total fixation duration significantly longer than all other patterns. Leopard and snakeskin patterns had total fixation durations that were significantly longer than the plain pattern. In Experiment 2, we employed a visual-search task where participants were required to find models wearing the various patterns in a setting of a crowded airport terminal. Participants detected leopard skin pattern and repetitive floral pattern significantly faster than two of the nonpatterned clothing styles. Our experimental findings support the hypothesis that repetition of specific visual features might facilitate target detection, especially those characterizing evolutionary important threats. Our findings that intricate, but nonthreatening repeating patterns can have similar attention-grabbing properties to animal skin patterns have important implications for the fashion industry and wildlife trade.

Attention is defined as the ability to prioritize ecological and biological items, such as objects in the environment or stored memories, by relevance and importance (Corbetta, Miezin, Dobmeyer, Shulman, & Petersen, 1990). In the visual system, even with a large field of vision, organisms focus attention on relatively small areas of the scene, which are analyzed in detail (Frintrop, 2006), providing for higher level processing such as object recognition. Visual attention is a high-demand process, being pulled in every direction from the diverse input afforded by our complex world. Due to the amount of complex perceivable sensory data in the environment, organisms are ecologically adapted to attend to what is relevant in order to behave in an effective manner and succeed in their environments (Frintrop, 2006; Wolfe & Horowitz, 2004).

A review by Carrasco (2011) defined visual attention to consist of three main types: spatial attention, object-based attention, and feature-based attention (FBA). Spatial attention refers to selectively processing one location over another and object-based attention is guided by recognizing the structure of the object. In human and nonhuman primates, FBA refers to the process of recognizing specific aspects of objects such as color, pattern, orientation, or direction of motion (Carrasco, 2011). Among these three types of attention, one would expect FBA to be most shaped by natural selection, because while locations and object structures can vary infinitely, there are often commonalities among objects in terms of features.

FBA can be selected for by natural selection to result in an attentional bias, which facilitate detection of threats, resources, mates, or other items that impact reproductive fitness. Attentional biases are present for recognizing various features of objects, which imbue them with higher salience, making them stand out relative to their surroundings. Pattern orientation is one such feature. For example, many primates capitalize on face and eye orientation of their conspecifics to focus their attention on objects of importance (Emery, 2000). Despite their poor visual acuity, African elephants have also been shown to pick up on body orientation and differentially signal to request food when the experimenter was facing the elephant as opposed to away from it (Smet & Byrne, 2014).

Another feature for which there is an attentional bias is direction of movement. Animals across taxa such as flies (Jablonski & Strausfeld, 2000), crabs (Oliva, Medan, & Tomsic, 2007), frogs (Yamamoto, Nakata, & Nakagawa, 2003), chickens (Evans, Macedonia, & Marler, 1993), monkeys (Schiff, Caviness, & Gibson, 1962), and humans (Vagnoni, Lourenco, & Longo, 2012) have been shown to recognize and respond to looming stimuli, where the stimuli are approaching them.

Similarly, there is an attentional bias for responding to patterns that are indicative of threats and this bias has been observed across animal taxa as well. Young African jewelfish instinctively dart away when confronted with horizontal patterns of two facing eyes (Coss, 1979). Lab-born California ground squirrel pups that have never been exposed to the wild are attentive when presented a snake-like pattern (Coss, 1991). Congruently in primates, the repetitive scales of snakes appear especially salient (Etting & Isbell, 2014; Kawai & He, 2016), bonnet macaques in Southern India exhibited greater alarm calling, greater frequency of flight, and a faster flight time in response to a spotted leopard model compared to a dark leopard model without a spotted pattern (Coss & Ramakrishnan, 2000).

Similarly, natural selection is thought to shape the evolution of visual system in humans too (Isbell, 2006). This evolved attentional bias persists in humans today, even among those living in a highly modified, often urban environment, where animal threats are no longer present and patterns signifying animal threats are no longer relevant. This is because the present design of animals is determined by historical sources of selection (Tooby & Cosmides, 1990). As such, the attentional bias for detecting threats in the environment can still be automatic triggers of attention (Tooby & Cosmides, 1990). It has been shown that even small portions of snakeskin and snake scales are sufficient for detection and recognition by vervet monkeys (Isbell & Etting, 2017). Animals combine the knowledge of animal patterns with that animal’s behavior to respond appropriately. For example, bonnet macaques in India spent a greater proportion of time monitoring and observing when presented with a patterned model of a constricting python but startled or ran a greater proportion of encounters when presented with a patterned model of a venomous cobra, reflecting the various types of threats these two species of snakes posed (Ramakrishnan, Coss, Schank, Dharawat, & Kim, 2005).

This automatic orientation of attention toward threat stimuli could be related to the stimuli’s associations with fear. This automatic activation of a fear response was tested by Öhman, Flykt, and Esteves (2001) who found that participants were consistently faster at locating fear relevant stimuli compared to fear irrelevant stimuli against fear relevant and fear irrelevant backgrounds. Penkunas and Coss (2013a) conducted a study on children and adults’ abilities to detect historically important animal threats. Findings from the study revealed that American children and adults were better at detecting potentially dangerous target animals in visual matrices of nondangerous animals (e.g., target snakes in arrays of lizards) compared with nondangerous animals in visual matrices of dangerous ones (e.g., target antelopes in arrays of lions), where the dangerous animals “popped out” in the matrix of nonthreatening distractors. These effects were found to be consistent when tested in urban and rural India where children may be more likely to encounter threatening animals (Penkunas & Coss, 2013b). Eye tracking research using similar visual arrays of snakes and lizards and lions and antelope provided additional support that dangerous animals can be visually salient (see Yorzinski, Penkunas, Platt, & Coss, 2014). In a related context, Vagnoni, Lourenco, and Longo (2012) tested participants with looming stimuli, measuring perceived time-to-collision with the stimuli, found similar results. Participants underestimated the time-to-collision for threatening stimuli, such as snakes and spiders, as opposed to nonthreatening images such as butterflies and rabbits.

As mentioned earlier, attentional biases are often based on commonalities among objects in terms of features. For example, natural selection for responding to looming stimuli has likely acted upon a simple principle of physics: The angular size of objects that are approaching will extend upon a larger area in the retina (Nalbach, 1990). We similarly propose that natural selection has shaped the attentional bias for visual patterns with repetitive features, particularly tessellation. Tessellated patterns are complex geometrical patterns and shapes that are repetitive and comprise of stripes, spots, crossing-over lines, and many edges with no gaps in between or overlap. The features of many threatening animals such as scorpion chitin, snakeskin, or leopard skin, often feature tessellation. We propose that these repetitive patterns are visually conspicuous against the nonrepetitive randomness of the larger environment, allowing for natural selection to shape the visual system to respond selectively to these repeating patterns (cf. Coss, 2003; Isbell, 2006) and guide the evolution of appropriate behavior in response to these threats.

From a historical perspective, these evolutionarily provocative patterns have also played a role in human clothing. Decorative clothing is thought to reflect and convey information regarding the wearer’s self-image, personality, or social status (Kaiser, 1997), and thus a desire to stand out and be noticed. Animal prints are often used for this purpose not just today but have been used for a large part of human history. Cultures varying widely temporally and geographically, from cave dwellers in Southern Africa tens of thousands of years ago to the Aztecs in Mesoamerica a few hundreds of years ago, have depicted humans and mythological creatures wearing various animal skins for ritualistic purposes (Jolly, 2002). Other innately provocative patterns and features have been similarly used. Forward facing eye-like patterns have been used across cultures in an apotropaic manner intended to divert gaze and protect the wearer from the “evil eye” (Coldiron, 2005; Sütterlin, 1989). In recent decades, patterns representing dangerous animals such as leopards, snakes, and crocodiles, among others, are commonly used in clothing and accessories. As a result, we considered clothing as a suitable medium upon which to test the effectiveness of various patterns in capturing attention.

While the evocative properties of evolutionarily important threats are well established, the premise of whether repetition is the key feature behind recognition of evolutionarily important threats has not been empirically tested. The current study aims to empirically test this premise by examining how human respondents react to pictures of models wearing clothing that bears different repeating and nonrepeating patterns including animal skin patterns. Based on the literature, animal skin patterns should be the most provocative, which is to say that they should capture attention faster and hold attention longer in comparison with other nonanimal skin patterns. However, because we hypothesize that the driver of this evolutionary bias is pattern repetition, we broaden our hypothesis to predict that repeating nonanimal print patterns on clothing should be equally as provocative as repeating animal print patterns on clothing. We hypothesize that both the animal skin patterns and the repeating nonanimal skin patterns should engender a shorter time to fixation and elicit longer fixation duration than the nonrepeating nonanimal skin patterns.

We present below two experimental studies to test this premise. In Experiment 1, we aimed to investigate attention bias toward the various categories of test stimuli. We anticipated that ecologically relevant patterns would lead to a longer fixation duration compared to ecologically irrelevant patterns. In Experiment 2, we aimed to investigate the idea that potentially threatening patterns “pop out” of the environment and into attentional focus relative to nonthreatening patterns. To examine pattern salience, we anticipated that participants engaged in the visual-search task would locate repetitive clothing patterns faster than nonrepetitive clothing patterns.

## Experiment 1

Experiment 1 was a free observation task where participants observed two pictures of the same model side by side on the screen wearing different patterns on clothing. The purpose of Experiment 1 was to evaluate whether certain patterns were more evocative than others, as evidenced by greater total fixation duration from participants preferentially spending more time looking at them. It was expected that ecologically relevant patterns would elicit greater total fixation duration compared to ecologically irrelevant patterns.

## Method

### Participants

Fifty-one students from the University of Nottingham Malaysia Campus took part in this experiment. Participants’ ages ranged between 18 and 31 years (*M* = 21.35, *SD* = 2.08 years) comprising of 9 males and 42 females. All participants had normal or corrected to normal vision.

### Ethics

The Science and Engineering Research Ethics Committee of the University of Nottingham Malaysia Campus provided ethics approval for this research under protocol number AZ220914.

### Apparatus and materials

**Figure 1.**
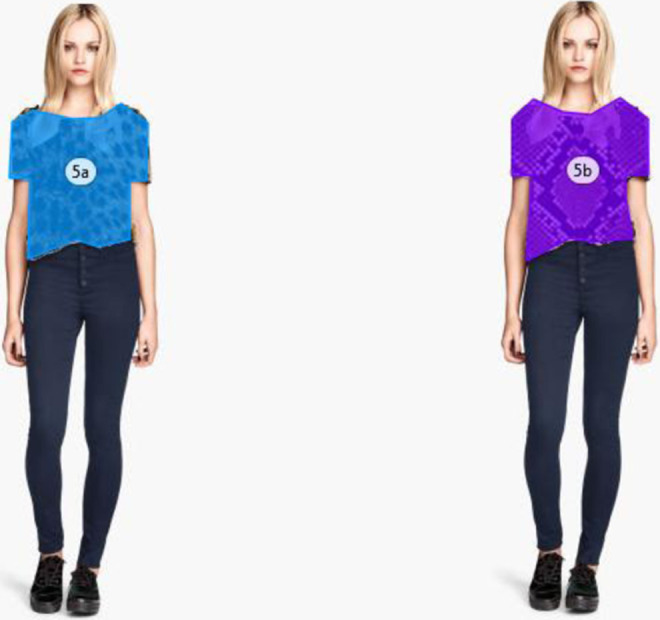
Examples of area of interests used in Experiment 1 where two patterns were presented side by side with participants instructed to observe freely.

We created stimuli consisting of four male and four female models in relaxed postures (Appendix A), each wearing six different clothing patterns; leopard (*Panthera pardus*) rosettes print, Southeast Asian reticulated python (*Python reticulatus*) snakeskin print, floral design, camouflage shapes, plain surface, and shiny surface (Appendix B). Each model was duplicated and the clothing pattern was edited for the six different designs using GNU Image Manipulation Program (GIMP) Version 2.8.2. In this experiment, trials composed of images of the same model in different clothing patterns placed side by side, in all possible combinations of the patterns. These combinations were randomized and counterbalanced. Therefore, for this experiment, participants responded to 120 trials of model–pattern combinations presented side by side. For each trial, an area of interest (AOI) was defined over each of the clothing patterns presented over which the eye tracker would monitor fixation (Figure 1). Hence, for each trial consisting of two models wearing different patterns side by side, two areas of interest were defined. The experiment was conducted using the Tobii Eye Tracker T120XL and the Tobii Pro Studio software (version 3.1.6), which were used to program the task. All stimuli were automatically adjusted to the eye tracker’s 1,280 × 1,024 pixel resolution display. The models displayed to the participants on the eye tracker measured 9.5 cm in height and 2.9 cm in width. Participants’ viewing distance to the screen was approximately 48 cm.

### Design

A within-participants design was used. The independent variable was type of pattern, consisting of six levels (leopard, snake, floral, camouflage, plain, and shiny). Although absent of surface texture, a shiny clothing feature was included because shiny objects have been shown to attract attention aesthetically (Coss, 1990; Coss, Ruff, & Simms, 2003; Meert, Pandelaere, & Patrick, 2014). The dependent variable was total fixation duration, measured in milliseconds (ms). Total fixation duration is the total amount of time that the participant fixates on an AOI on the image while it appears on the screen.

### Procedure

**Figure 2.**
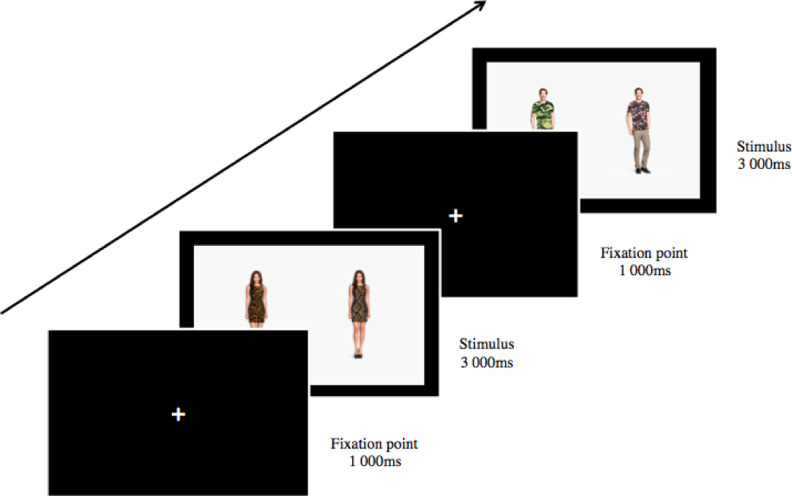
Diagrammatic representation of the experimental procedure for experiment.

After being presented with and signing an informed consent form, they were briefed on the task. After a calibration procedure with the Tobii Eye Tracker, the experiment began, which consisted of one block of 120 randomized and counterbalanced trials being made up of all combinations of patterns for all models. Each trial consisted of a duration of 4,000 ms, during which time a white fixation cross would first appear in the center of a black background for 1,000 ms followed by a pair of pattern-wearing models presented side by side for another 3,000 ms. After this, the next trial would begin automatically, starting with the fixation cross on a black background for 1,000 ms and then a new pair of pattern-wearing models presented side by side for 3,000 ms. This continued until all 120 trials were completed. The experiment lasted a total of eight minutes (120 trials of 4 s each). Participants were instructed to look at the fixation cross when it was present on screen but otherwise look at the screen freely wherever they chose. See Figure 2 for a diagrammatic representation of the experimental procedure.

## Results

A repeated measures analysis of variance (ANOVA) was conducted to investigate whether there was a main effect of pattern (leopard, snake, floral, camouflage, plain, and shiny) on the total duration of visual fixation. Mauchly’s test indicated the assumption of sphericity had been violated for pattern, χ^2^(14) = 26.181, *p* = .025; therefore, degrees of freedom for pattern were adjusted using Huynh–Feldt estimates of sphericity (∊ = .850). Results from the ANOVA found a significant main effect of pattern; *F*(4.7, 234.7) = 12.985, *p* < .001.

**Figure 3.**
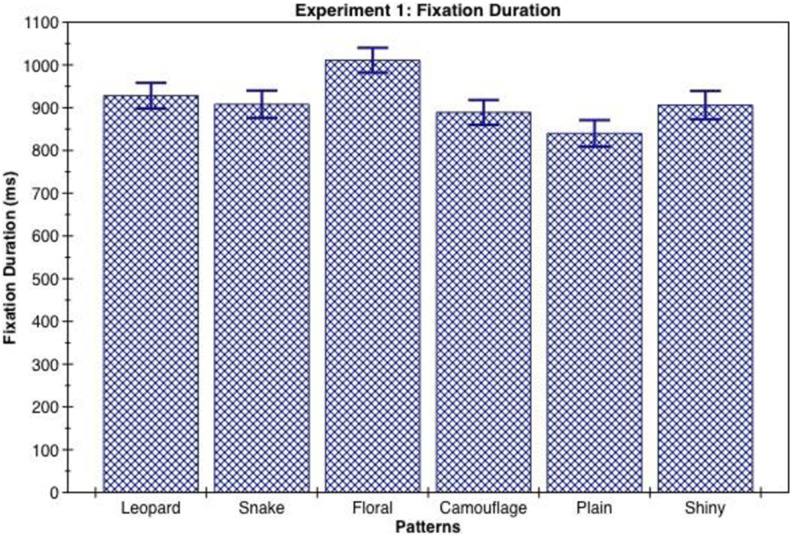
Mean and standard error of total fixation duration for clothing patterns measured in milliseconds.

Pairwise comparisons showed that the floral pattern obtained reliably greater fixation duration than all of the other clothing designs used, while the leopard skin, snakeskin, shiny, and camouflage patterns obtained reliably greater fixation duration than the plain clothing (see Table 1 and Figure 3).

**Table 1.** Mean and 95% Confidence Interval (CI) of Total Fixation Duration for Clothing Patterns Measured in Milliseconds (ms).

Fixation Duration (ms)	Patterns	Mean	95% CI
	Leopard	928	[868, 989]
	Snake	908	[844, 972]
	Floral	1,011	[953, 1,068]
	Camouflage	889	[830, 948]
	Plain	840	[777, 903]
	Shiny	906	[840, 972]

*Note.* Significant differences: ***p < .001 floral > all others, leopard > plain. **p < .01 snake > plain, shiny > plain. *p < .05 camouflage > plain.

## Discussion

In this experiment, the animal skin patterns of leopard rosettes and snake-scale shapes elicited greater viewing time from participants as expected. However, we also found that, among the entire set of clothing patterns, the floral pattern engendered the longest visual fixation. We propose that this might be a result of an attentional bias where participants were evaluating the intricate and complex aspect of the floral print. Finally, as anticipated, we found that the plain pattern was the least evocative among the patterns tested.

## Experiment 2

Experiment 2 was a visual-search task where participants had to locate models that they were familiar with from Experiment 1 in an image of a crowded airport terminal. The purpose of Experiment 2 was to evaluate whether certain patterns were more evocative than others, as evidenced by a shorter time to first fixation from participants noticing them faster. It was expected that repetitive patterns, particularly ecologically relevant patterns, would elicit a shorter time to first fixation compared to nonrepetitive patterns.

## Method

### Participants

Forty-five students from the University of Nottingham Malaysia Campus took part in this study. Participants’ ages ranged between 18 and 31 years (*M* = 21.46, *SD* = 2.10 years) comprising of 8 males and 37 females. All participants had normal or corrected to normal vision. Participants taking part in this experiment had also taken part in Experiment 1 and were therefore familiar with the appearance of the models and patterns at the start of the experiment. Both experiments were conducted during the same session, with a short break provided for participants between Experiments 1 and 2.

### Apparatus and materials

**Figure 4.**
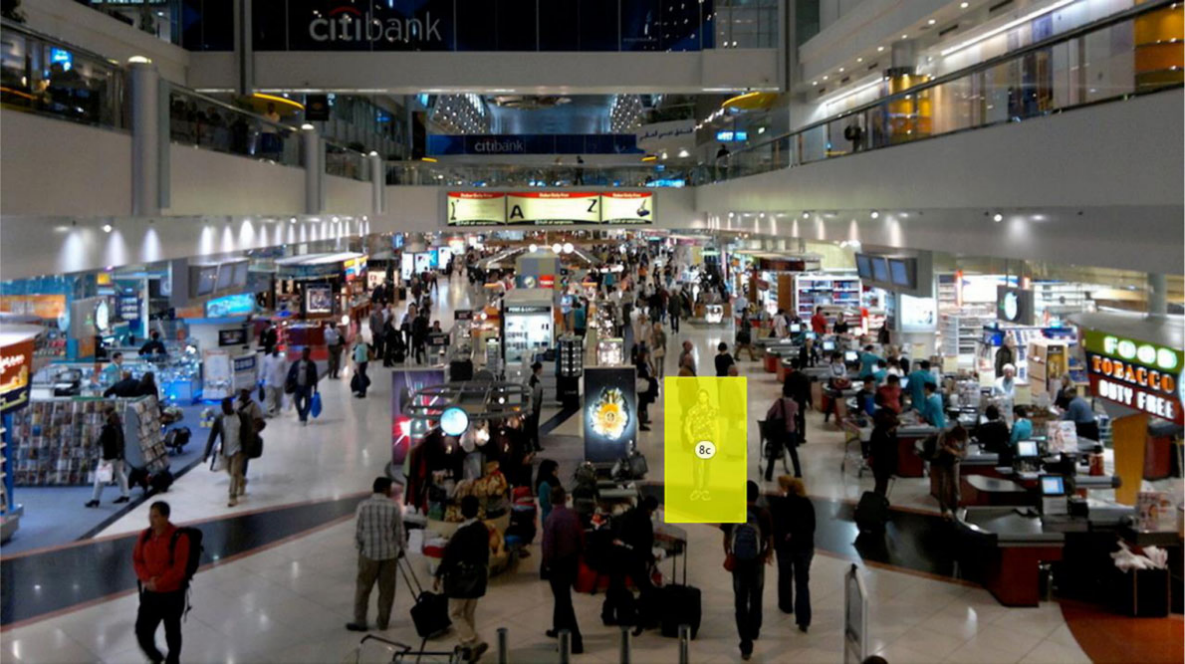
Examples of area of interest used in Experiment 2 where participants had to find the target model in the crowded airport terminal setting.

We created stimuli consisting of six male and six female models (Appendix A), each wearing six different clothing patterns; leopard (*P. pardus*) rosettes print, Southeast Asian reticulated python (*P. reticulatus*) snakeskin print, floral print, camouflage print, plain print, and shiny print (Appendix B). Each model was duplicated and the clothing pattern was edited for the six different designs using GIMP Version 2.8.2. In each of the 72 trials, a model wearing a pattern was placed in an image of a crowded Dubai International Airport terminal (refer to Appendix C). The target models were placed in one of nine different locations on the terminal image, from front to back and left to right. The size of the target varied depending on whether it appeared in the front, middle, or back of the picture. The dimensions were 4.2-cm height × 0.9-cm width for the front, 3.0-cm height × 0.8-cm width for the middle, and 2.0-cm height × 0.5-cm width for the back. Participants’ viewing distance to the screen was approximately 48 cm. The locations were randomized and counterbalanced for the experiment, so all of the patterns were observed on multiple models at each possible location to control for size effects. For each trial, an AOI was defined using rectangular AOIs (see Figure 4 for an example of AOIs) over the target model, with a single AOI being present during each trial.

The experiment was conducted using the Tobii Eye Tracker T120XL and the Tobii Pro Studio software, which was used to program the task. All stimuli were automatically adjusted to the eye tracker’s 1,280 × 1,024 pixel resolution display.

### Design

Similar to Experiment 1, a within-participants design was used. The independent variable was type of pattern, consisting of six levels (leopard, snake, floral, camouflage, plain, and shiny). The dependent variable was the time to first fixation (fixation latency) measured in ms. The time to first fixation was the period between the appearance of the image on the screen and when the participant shifted his or her attention by fixating the image AOI.

### Procedure

**Figure 5.**
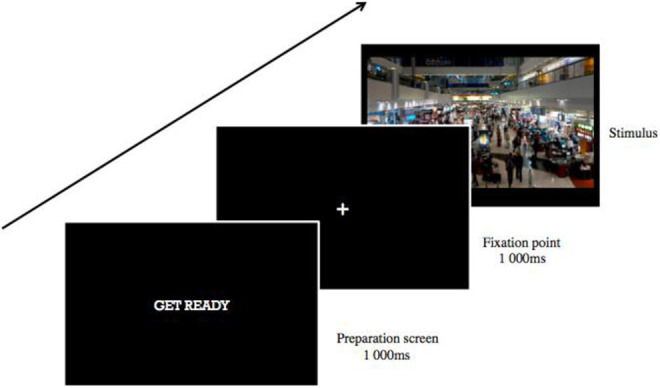
Diagrammatic depiction of a single trial for the search task in Experiment 2.

Participants were briefed on the task, and informed consent was obtained. After a calibration procedure, the experiment began, which consisted of one block of 72 trials. Each trial consisted of an image obtained from Google Images of a crowded terminal building from Dubai International Airport with a model wearing one of the clothing patterns in one of nine locations on the image (top right, top center, top left, middle right, middle center, middle left, bottom right, bottom center, and bottom left). The same background image was used in all trials with only the location of the models being varied. The size of the model wearing the pattern was adjusted to match the size of other individuals in the image around the target model. The location of the models on the image was randomized and counterbalanced across the 72 trials. Each trial first began with a preparation screen with the text “GET READY” in white color font in the center of a black background for 1,000 ms. This was then followed by a white fixation cross appearing in the center of a black background for another 1,000 ms, after which the airport terminal image was presented. The participants had been instructed to examine the scene until they locate one of the models they had seen previously in Experiment 1. When they had located the target, they were instructed to provide a manual response by clicking on the mouse in order to proceed to the next trial. See Figure 5 for a diagrammatic representation of the experimental procedure.

## Results

A repeated measures ANOVA was conducted, measuring the fixation latency in the visual-search task, to investigate whether there was main effect of clothing pattern (leopard, snake, floral, camouflage, plain, and shiny). Mauchly’s test of sphericity indicated the assumption of sphericity had been violated, χ^2^(14) = 41.175, *p* < .001; therefore, degrees of freedom were adjusted using Greenhouse–Geisser estimates of sphericity (∊ = .728). Results from the ANOVA found a significant main effect of pattern; *F*(3.6, 160.1) = 3.068, *p* = .022.

**Figure 6.**
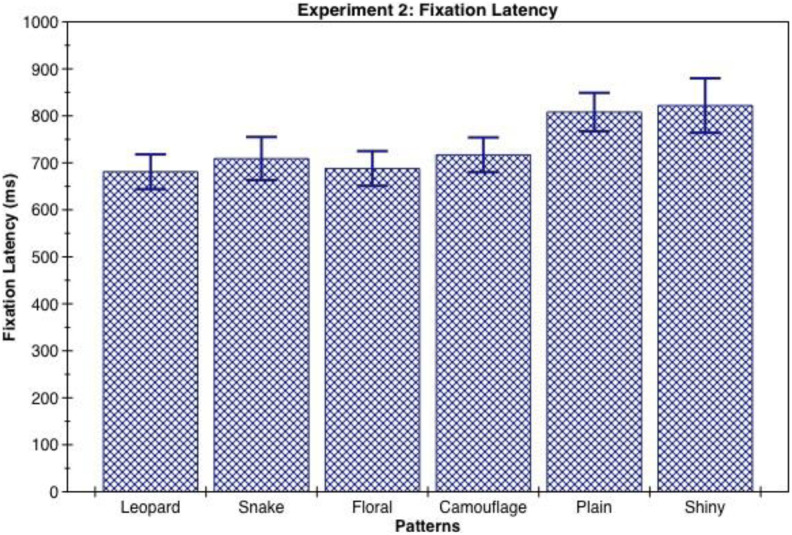
Mean and standard error of fixation latency for clothing patterns in the visual-search task measured in milliseconds.

Pairwise comparisons showed that leopard skin, floral print, and camouflage patterns were detected reliably faster than the plain and shiny clothing designs (see Table 2 and Figure 6).

**Table 2.** Mean and 95% Confidence Interval (CI) of Fixation Latency for Clothing Patterns in the Visual-Search Task Measured in Milliseconds (ms).

Fixation Latency (ms)	Patterns	Mean	95% CI
	Leopard	697	[626, 767]
	Snake	725	[637, 813]
	Floral	703	[635, 772]
	Camouflage	726	[652, 799]
	Plain	815	[730, 899]
	Shiny	833	[716, 950]

*Note.* Significant differences: **p < .01 floral < plain. *p < .05 floral < shiny, leopard < plain, leopard < shiny, camouflage < plain, camouflage < shiny.

## Discussion

In this experiment, both the animal skin pattern of leopard and the repetitive floral pattern were located significantly faster than the plain and shiny patterns. However, the snakeskin pattern did not show the anticipated effect. We propose that this might be a result of the species of snakeskin used, which was prone to pattern blending when viewed at a distance and thus resulted in higher variation in fixation of the target by participants leading to a nonsignificant comparison. Surprisingly, the camouflage pattern was located significantly faster than the plain and shiny patterns, and we propose that this was due to the high contrast of the pattern that made it stand out in the context of an airplane terminal where typical pattern blending of the camouflage design could not occur.

## General Discussion

Our findings support the hypothesis that repetition of specific visual features might facilitate target detection, especially those characterizing evolutionary important threats. In Experiment 1, the animal skin patterns of leopard rosettes and snake-scale shapes elicited significantly higher total fixation duration than the plain pattern. This is consistent with previous findings from literature that animal skin patterns are evocative (Coss, 1991; Coss & Ramakrishnan, 2000; Öhman, Flykt, & Esteves, 2001; Penkunas & Coss, 2013a, 2013b; Tooby & Cosmides, 1990; Yorzinski et al., 2014).

**Figure 7.**
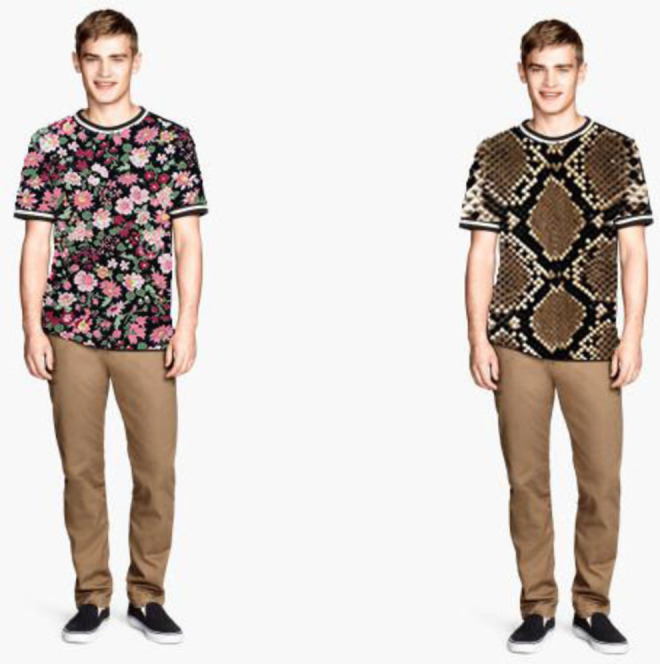
Sample stimulus from Experiment 1, with a side-by-side comparison of the floral and snake print patterns.

Contrary to prediction, however, we also found that, among the entire set of clothing patterns, the floral pattern engendered the longest visual fixation. This result might reflect an attentional bias in which participants were evaluating the intricate and complex aspects of the floral prints (see Figure 7). This argument is consistent with the finding that the least decorative plain pattern sustained the reliably shortest attention among the set of clothing patterns. Our findings that the leopard and snake animal patterns were less evocative than the floral pattern do not support our hypothesis. It may be that the floral pattern was more evocative as a result of familiarity (Hekkert, Snelders, & van Wieringen, 2003), but this is unlikely because the floral pattern that we used was a very intricate and repeating pattern that is not commonly observed in floral prints. Another explanation is that the floral patterns were preferred because the animal skin patterns engendered selective avoidance due to their provocativeness (Bradley, Costa, & Lang, 2015), but this is also unlikely because the animal skin patterns were selectively preferred compared to the plain pattern. A more likely explanation of our findings are that the ecologically important properties of these patterns are not relevant when presented in the decorative context of clothing, in a situation where there is no apparent threat that can be evaluated by sustained fixation. The floral pattern used in our experiment may have had enhanced visual appeal as a result of it having a combination of two salient features: aesthetically pleasing flowers and attention-grabbing repetitiveness.

In Experiment 2, when participants were required to locate the pattern in a visual-search task, the animal skin pattern of leopard and the repetitive floral print patterns exhibited nearly equal mean fixation latency from participants. They both were located significantly faster than the plain and shiny patterns. The speed of detection of the floral pattern supports the hypothesis that the repetitive properties of the floral pattern enhanced its conspicuousness. A complementary explanation is that by introducing artificial repetitiveness into a pattern made up of flowers, the floral pattern evoked a sense of danger typically associated with snakeskin, or other repetitive patterns found on animals.

While the snake and camouflage patterns had nearly equal mean fixation latencies, the camouflage pattern was detected significantly faster than the plain and shiny patterns, whereas there was no significant effect toward faster detection of the snake pattern compared to the plain and shiny patterns (*p* = .10). We used the Southeast Asian reticulated python for the snakeskin pattern because it is a commonly used pattern for snakeskin designs and we expected the repeating diamond shapes to be very provocative. The Southeast Asian reticulated python is a known predator of humans (Headland & Greene, 2011), likely dating back to the late-Pleistocene when modern humans first settled Southeast Asia. However, the fine crosshatched snake scales are hard to resolve when viewed at a distance (see Kawai & He, 2016), leaving only the larger diamond clusters framing the snake scales visible as the predominant feature for capturing attention (Figure 7). This is a consequence of pattern blending where surfaces of black and white squares or black and white spotted surfaces appear to be of an even gray tone when viewed from a distance (Mottram, 1915). This may have resulted in the relatively higher variation in visual fixation of the target image by participants for the snakeskin pattern and the nonsignificant effect in comparison with the plain and shiny patterns.

On the other hand, the surprising evocativeness of the camouflage pattern compared to the plain and shiny patterns could be attributed to the complexity of the camouflage design and the high contrast and brightness of the pattern used relative to the surroundings. While the camouflage pattern may be random and blend into a natural setting with similar backgrounds, when used in our experimental scenarios, particularly in the setting of an airport terminal mall in Experiment 2, it stood out substantially relative to its surroundings without the consistency necessary for pattern blending to occur (see Mottram, 1915).

Our findings could have important implications for wildlife trade. Animal skins and furs are traded to produce clothing, shoes, bags, decorations, and other items (Broad, Mulliken, & Roe, 2003). This trade consists of illegally (such as leopard skins) and legally (such as snakeskins) traded products (Broad et al., 2003; Zhou & Jiang, 2004). From the late-1990s onward, there was a surge in the use of animal skins in clothing in China, with the previously uncommon trade being driven more by fashion than by tradition (World Bank, 2010). The use of animal skins being driven by fashion in clothing and decoration may be to reflect and convey information regarding the wearer’s self-image, personality, or social status (Kaiser, 1997), and thus a desire to stand out and be noticed.

However, our findings indicate that animal skin patterns do not carry their ecologically provocative properties through to clothing. Our findings suggest that an artificially created repeating pattern or a high contrast pattern can carry equally provocative properties through to clothing. For example, the floral pattern held attention longer than any of the animal skin patterns; the camouflage pattern showed faster detection when compared against a plain pattern in a way that the snakeskin pattern did not. Our results show that in the context of clothing fashion trends could be driven toward these patterns as being more effective in reflecting wearers’ desire to stand out and be noticed.

In conclusion, our findings support our broader hypothesis that pattern repetition in clothing prints is a key visual feature that captures and maintains attention. Rapid fixation of repetitive clothing patterns is similarly affected by pattern complexity in which higher visual contrast captures attention much more effectively than less contrasting shiny or plain clothing patterns. Future research on pattern repetition will address the unanticipated contextual properties of attentional biases to intricate but nonthreatening patterns.

## Figures and Tables

**Figure A1.**
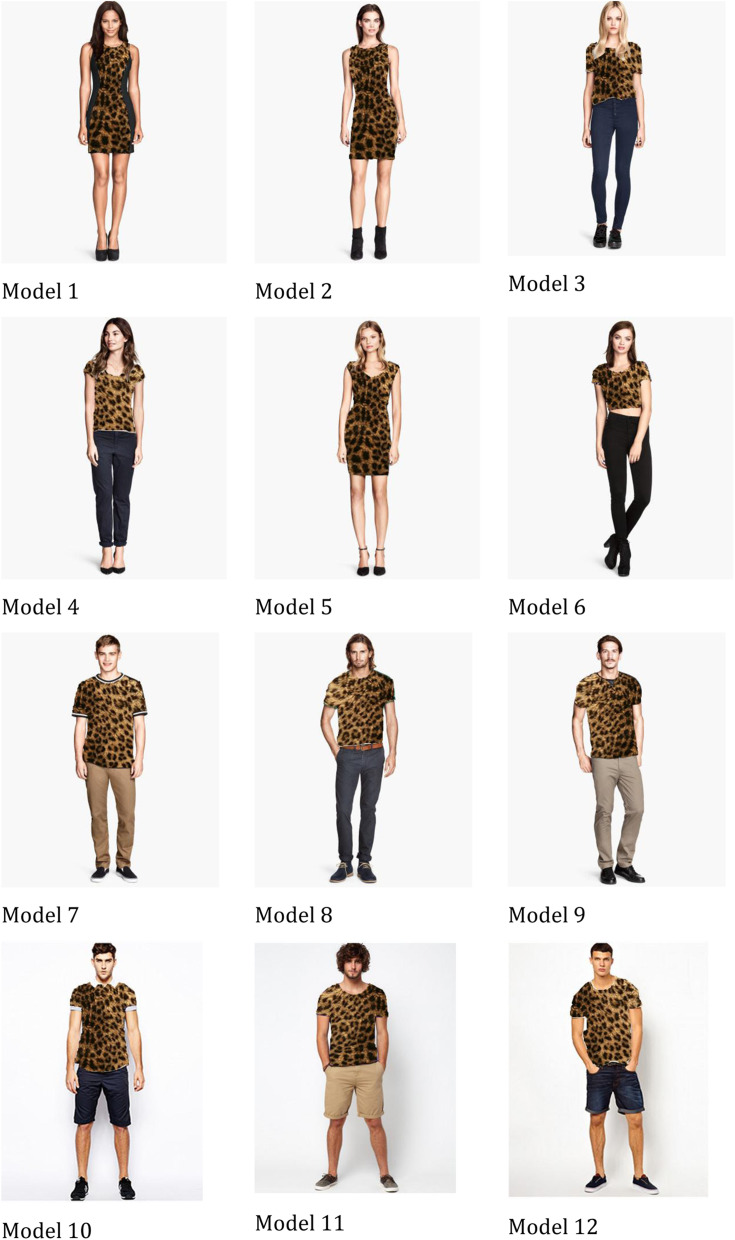
Models.

**Figure B1.**
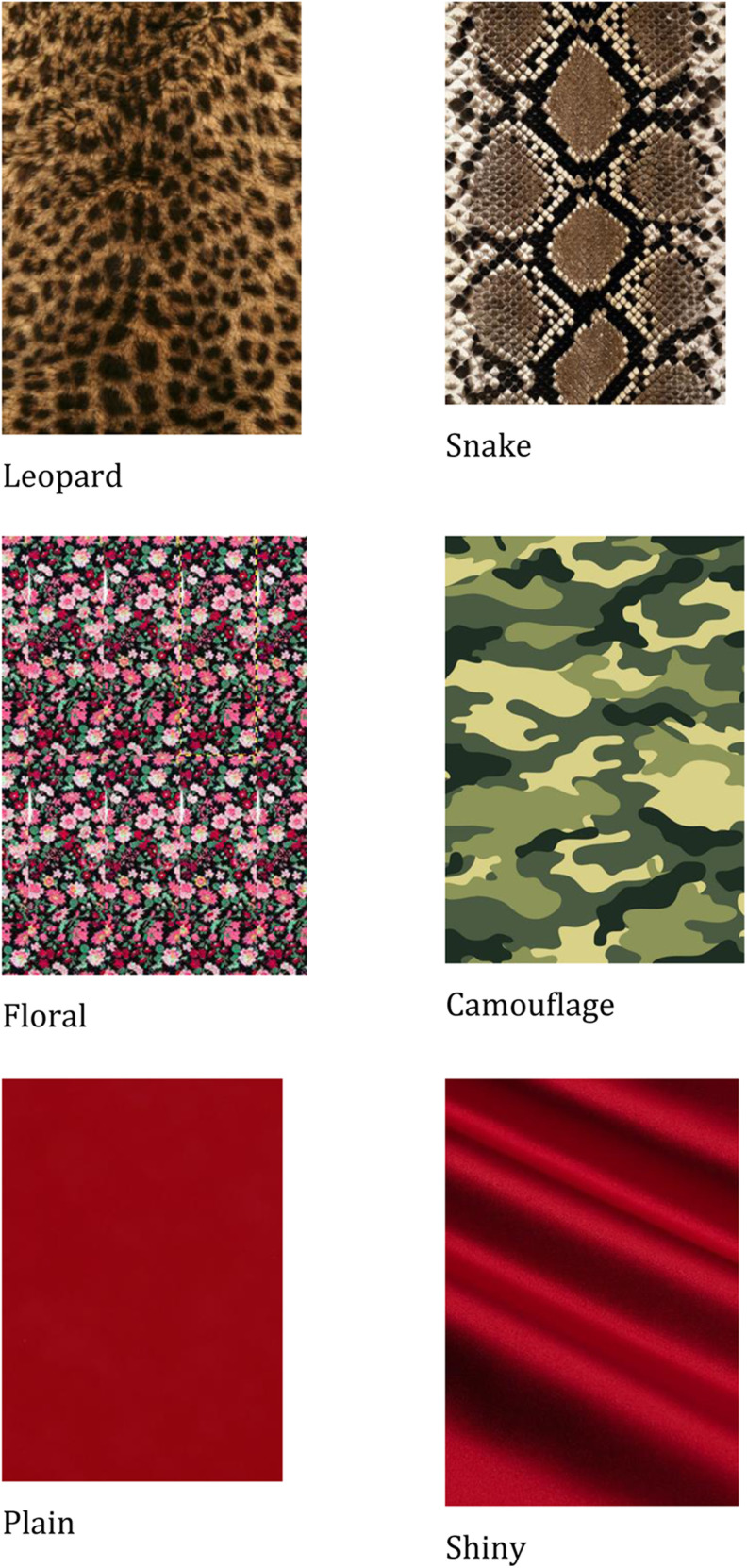
Patterns.

**Figure C1.**
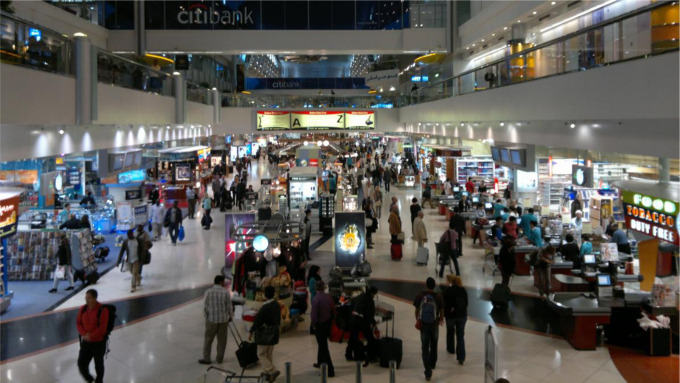
Experiment 2 visual-search task scene.

## Appendix C

Dubai International Airport Setting.
